# Plantaricin BM-1 decreases viability of SW480 human colorectal cancer cells by inducing caspase-dependent apoptosis

**DOI:** 10.3389/fmicb.2022.1103600

**Published:** 2023-01-04

**Authors:** He Wang, Junhua Jin, Xiaona Pang, Zheng Bian, Jingxin Zhu, Yanling Hao, Hongxing Zhang, Yuanhong Xie

**Affiliations:** ^1^Beijing Laboratory of Food Quality and Safety, Beijing Key Laboratory of Agricultural Product Detection and Control of Spoilage Organisms and Pesticide Residue, College of Food Science and Engineering, Beijing University of Agriculture, Beijing, China; ^2^Department of Nutrition and Health, Ministry of Education and Beijing Government, Beijing, China

**Keywords:** apoptosis, bacteriocin, colorectal cancer, plantaricin BM-1, transcriptomics

## Abstract

Plantaricin BM-1 is a class IIa bacteriocin produced by *Lactobacillus plantarum* BM-1 that has significant antimicrobial activity against food-borne bacteria. In this study, a cell proliferation assay and scanning electron microscopy were used to detect changes in the viability of SW480, Caco-2, and HCT-116 colorectal cancer cells treated with plantaricin BM-1. We found that plantaricin BM-1 significantly reduced the viability of all colorectal cancer cell lines tested, especially that of the SW480 cells. Scanning electron microscopy showed that plantaricin BM-1 treatment reduced the number of microvilli and slightly collapsed the morphology of SW480 cells. Fluorescence microscopy and flow cytometry demonstrated that plantaricin BM-1 induced apoptosis of SW480 cells in a concentration-dependent manner. Western blotting further showed that plantaricin BM-1-induced apoptosis of SW480 cells was mediated by the caspase pathway. Finally, transcriptomic analysis showed that 69 genes were differentially expressed after plantaricin BM-1 treatment (*p* < 0.05), of which 65 were downregulated and four were upregulated. The Kyoto Encyclopedia of Genes and Genomes enrichment analysis showed that expression levels of genes involved in the TNF, NF-κB, and MAPK signaling pathways, as well as functional categories such as microRNAs in cancer and transcriptional misregulation in cancer, were affected in SW480 cells following the treatment with plantaricin BM-1. In conclusion, plantaricin BM-1 induced death in SW480 cells *via* the caspase-dependent apoptosis pathway. Our study provides important information for further development of plantaricin BM-1 for potential applications in anti-colorectal cancer.

## 1. Introduction

The colorectal cancer (CRC) is the third most common cancer worldwide and ranks second among the causes of global cancer mortality (Bray et al., 2018). Common colon cancer cell lines, such as Caco-2, SW480, HCT116, etc., are often used *in vitro* for preliminary screening of anticancer drugs and mechanism research. Besides, existing anticancer drugs kill cancer cells, but also have toxic side effects on normal cells (Schlichtig et al., 2019). Therefore, it is very meaningful to search for anticancer components with high efficacy and low toxicity from natural products. Generally, these natural products kill cancer cells by inhibiting cell proliferation and inducing apoptosis or necrosis (Cui et al., 2020). Apoptosis is a genetically controlled method of programmed cell death that does not induce an inflammatory response (Xu et al., 2019). Necrosis is usually associated with swelling and loss of membrane integrity as a result of physical injury that does not involve the active participation of the cell (Zhang et al., 2019). In contrast to necrosis, several morphological features, including chromatin condensation, cell contraction, nuclear fragmentation, and membrane permeability, commonly occur during apoptosis (Chen et al., 2018). The core executioners of apoptosis are the caspases family proteases. Among them, Caspase-3 is the ultimate executor of cell apoptosis (Yang et al., 2019). Cleaved caspase-3 disrupts anti-apoptotic proteins and releases pro-apoptotic C-terminal fragments (Asadi et al., 2022). Poly (ADP-ribose) polymerase (PARP-1) is the main substrate of caspase-3 and a key enzyme for detecting DNA damage. Cleaved caspase-3 triggers PARP-1 cleavage. The cleaved PARP-1 then accesses chromatin *via* endonucleases to induce apoptosis (Zhou et al., 2021).

Bacteriocins are short-chain peptides produced by bacterial ribosomes that are commonly used as antimicrobial agents in food and pharmaceuticals (Soltani et al., 2021). Bacteriocins are usually studied for their antibacterial effect, but they are also increasingly considered potential anticancer drugs (Baindara et al., 2018; Yaghoubi et al., 2020; Cesa-Luna et al., 2021). For example, colicins (A, E1, U, and E3; Arunmanee et al., 2021) and pediocin PA-1 (Villarante et al., 2011) were shown to inhibit HT29 CRC cell viability *in vitro*. In addition, antimicrobial peptides M2163 and M2386 induced apoptosis of SW480 CRC cells by enhancing cell membrane permeability (Tsai et al., 2015). Furthermore, the antimicrobial peptide KL15 obtained *via in silico* modification of the sequences of bacteriocins m2163 and m2386 damaged the cell membrane of SW480 cells by forming pores, thus inducing necrosis. Notably, KL15 was not cytotoxic to normal human mammary epithelial cells (Mejía-Caballero et al., 2021). In addition, nisin was shown to induce apoptosis in SW480 CRC cells through the intrinsic apoptotic pathway (Jaye et al., 2022). Microcin E492, a bacteriocin produced by the *Klebsiella pneumoniae* strain RYC492, showed antitumor activity against cancer cells and induced apoptosis by activating the caspase pathway at low concentrations and necrosis at high concentrations (Varas et al., 2020). However, the exact mechanism of action of bacteriocins against CRC cells has not yet been fully elucidated.

Plantaricin BM-1 is a novel class IIa bacteriocin produced by *Lactobacillus plantarum* BM-1, a probiotic isolated from a traditional natural fermented meat product. Our previous research showed that this bacteriocin has significant inhibitory activity against food-borne pathogenic bacteria, such as *L. monocytogenes*, *E. coli*, and *Salmonella* (Zhang et al., 2013), so it can be used to develop natural bio-preservatives. In the present study, we investigated whether plantaricin BM-1 has anticancer activity against SW480 CRC cells. We showed that plantaricin BM-1 induced apoptosis in SW480 CRC cells through the caspase-mediated pathway. Moreover, transcriptomic analysis was performed to provide detailed information on the potential molecular mechanisms of plantaricin BM-1 action against SW480 cells. This study provides an experimental basis for further development of plantaricin BM-1 as an anti-colorectal cancer compound.

## 2. Materials and methods

### 2.1. Preparation of plantaricin BM-1

A two-step method was used to purify plantaricin BM-1, as described in our previous study (Zhang et al., 2013). Briefly, *Lactobacillus plantarum* BM-1 was cultured in de Man, Rogosa, and Sharpe (MRS) broth at 37°C for 12 h and centrifuged at 4°C for 10 min at 10,000 rpm to collect the supernatant. Plantaricin BM-1 was purified *via* pH-mediated cell adsorption–desorption and cation-exchange chromatography, freeze-dried, and stored at −80°C.

### 2.2. Cell culture and culture conditions

SW480 (ATCC CCL-228), Caco-2 (ATCC HTB-37), and HCT-116 (ATCC CCL-247) cell lines were obtained from the Cell Resource Center of the Institute of Basic Medical Sciences (Chinese Academy of Medical Sciences, Beijing, China). The normal colonic epithelial cell line NCM460 (INCELL CVCL_0460) was obtained from iCell Bioscience, Inc. (Shanghai, China). SW480 and HCT-116 cells were cultured in Leibovitz’s L-15 medium (Gibco, New York, NY, United States) and Iscove’s Modified Dulbecco’s medium (Gibco) containing 10% fetal bovine serum (Gibco), respectively. Caco-2 and NCM460 cells were grown in RPMI-1640 medium (Gibco) containing 10% fetal bovine serum. Caco-2, HCT-116, and NCM460 cells were incubated in a humidified atmosphere of 95% air/5% CO_2_, whereas SW480 cells were not exposed to additional CO_2_. All cells were cultured in a CO_2_ incubator (HF90; Shanghai Lishen Scientific Equipment Co. Ltd., Shanghai, China) at 37°C.

### 2.3. Cell viability assay

Cell Counting Kit-8 (CCK-8 kit; Dojindo, Kumamoto, Japan) was used to evaluate the effect of plantaricin BM-1 on cell viability. Briefly, the cells were seeded at a density of 1 × 10^4^ cells/well in a 96-well plate 24 h before the addition of plantaricin BM-1. After overnight incubation, the medium was replaced by a fresh medium containing different concentrations of plantaricin BM-1 (181, 363, 726, 1,451, and 2,902 μg/ml, diluted with complete medium), and the cells were cultured. The medium was discarded after 1 h of reaction period. Then, 100 μl of the medium and 10 μl of the CCK-8 kit reagent were added to each well, and the plates were incubated at 37°C for 1 h. Cells treated with 100 μl of complete medium were used as the negative control. The final absorbance was measured at 450 nm using a microplate reader (ELx808; BioTek, Winooski, VT, United States). The following formula was used to assess viability: viability ratio (%) = [(absorbance of the experimental well − blank well absorbance)/(control well absorbance − blank well absorbance)] × 100%. Six biological replicates were used for each treatment. The IC_50_ value was estimated by viability rates and calculated using the logit of probit regression in SPSS 19.0.

### 2.4. Scanning electron microscopy

Approximately 2 × 10^5^ SW480 cells were seeded on several 0.17-mm glass coverslips (Solarbio, Beijing, China) in a six-well plate (Corning, New York, NY, United States). After 24 h, the growth medium was replaced with the medium containing different concentrations of plantaricin BM-1 (1/2 × IC_50_, 1 × IC_50_, or 2 × IC_50_), and the cells were incubated for 1 h. Cells not exposed to plantaricin BM-1 were used as the negative control. Cells were washed with ice-cold phosphate-buffered saline (PBS; Solarbio) and fixed in 2.5% glutaraldehyde (Solarbio) for 2 h at room temperature. The cells were prepared for Scanning electron microscopy (SEM) analysis according to conventional methods. After the cells were dehydrated with 50, 70, 90, and 100% ethanol, they were frozen overnight. The samples were placed on a specimen holder and sputtered with 20-nm gold onto an ion coater. An ultra-high resolution scanning electron microscope (SU8010; Hitachi, Tokyo, Japan) was used to image the specimens.

### 2.5. Fluorescence microscopic analysis of apoptosis

SW480 cells (4 × 10^5^ cells) were seeded in a 60-mm dish (Corning) and cultured for 24 h. After treatment with plantaricin BM-1 concentrations of 0, 1/2 × IC_50_, 1 × IC_50_, or 2 × IC_50_ for 1 h, cells were directly incubated with 10 μg/ml Hoechst 33342 (Solarbio) at 37°C for 10 min to stain for the DNA. After staining, the cells were washed with ice-cold PBS and examined under a fluorescence microscope (XD; Sunny Optical Technology Co. Ltd., Hong Kong, China) at 100 and 200× magnifications to observe chromatin condensation.

### 2.6. Flow cytometry analysis of apoptosis

SW480 cells were seeded at a density of 1 × 10^5^ cells/ml in six-well plate and treated with plantaricin BM-1 (0, 1/2 × IC_50_, 1 × IC_50_, or 2 × IC_50_) for 1 h. The cells were then harvested *via* trypsinization (Solarbio). The harvested cells were washed twice with PBS. An annexin V-FITC/PI kit (4A Biotech, Suzhou, China) was used for double staining. Apoptotic cells were analyzed using flow cytometry (NovoCyte; ACEA Biosciences, San Diego, CA, United States). Based on the flow cytometry results, apoptotic cells were mainly composed of early (Annexin V-FITC^+^/PI^−^) and late (Annexin V-FITC^+^/PI^+^) apoptotic cells.

### 2.7. Western blotting analysis

To estimate the influence of plantaricin BM-1 on the cleavage of caspase-3 and PARP-1, SW480 cells were treated with the complete medium (negative control), different concentrations of plantaricin BM-1 (1/2 × IC_50_, 1 × IC_50_, and 2 × IC_50_), or with 5-fluorouracil (5-FU; 757.9 μg/ml; positive control), and incubated for 1 h. Then, the cells were lysed and proteins were extracted using a Protein Extraction Kit (GenePool, Beijing, China). The proteins were using an SDS-PAGE Gel Kit (GenePool), containing 10% (cleaved PARP-1) or 15% (cleaved caspase-3) separating gel and 5% stacking gel, and then transferred to a polyvinylidene fluoride (PVDF) membrane (GenePool) with a pore size of 0.22 μm for 0.5 h (cleaved PARP-1) or 2 h (cleaved caspase-3). After the transfer, the PVDF membrane was immersed in a milk-blocking buffer (GenePool)/bovine serum albumin-blocking buffer (GenePool) for 1 h to block non-specific binding. The membrane was incubated at 4°C overnight with anti-PARP (Bioss, Beijing, China, No. bs2138R), anti-cleaved caspase-3 (Cell Signaling Technology, Danvers, MA, United States, No. 9664), and anti-β-actin (Abcam, Cambridge, United Kingdom, No. ab6276) primary antibodies diluted with 1% BSA/5% milk. The membrane was washed with TBST (GenePool) three times and then incubated with a milk-blocking buffer containing either goat anti-rabbit IgG-HRP (Abcam, Cambridge, United Kingdom) or goat anti-mouse IgG-HRP (Abcam, Cambridge, United Kingdom) diluted at 1:5,000, and gently shaken for 50 min at room temperature. After washing with TBST four times, the PVDF membrane was immersed in an ECL Plus Western Blot Kit color-developing solution (GenePool) for 1 min and then exposed and developed in a dark room. Quantity One V.4.6.2 software was used to analyze gray values.

### 2.8. RNA-sequencing analysis and gene set enrichment analysis

RNA sequencing was performed on plantaricin BM-1-treated (D group) or complete medium-treated (C group) cell samples based on the BMKCloud platform.^1^ And three biological replicates were used for D group (D1, D2, and D3) and C (C1, C2, and C3). In brief, SW480 cells (1 × 10^5^ cells/ml) were treated with 1 × IC_50_ concentration of plantaricin BM-1 for 1 h, trypsinized to collect cells, and rinsed three times with PBS. Total RNA was extracted using the TRIzol reagent at 4°C. RNA concentration and purity were measured using a NanoDrop 2000 spectrophotometer (Thermo Fisher Scientific, Waltham, MA, United States). RNA integrity was assessed using the RNA Nano 6000 Assay Kit on an Agilent Bioanalyzer 2100 system (Agilent Technologies, Santa Clara, CA, United States). RNA samples that met the conditions of 2.5 ≥ OD_260_ nm/OD_280_ nm ≥ 1.7, 2.5 ≥ OD_260_ nm/OD_230_ nm ≥ 0.5, and RNA integrity number ≥ 7.0 were used for subsequent experiments. Sequencing libraries were generated using the NEBNext UltraTM RNA Library Prep Kit for Illumina (New England Biolabs, Ipswich, MA, United States) following the manufacturer’s recommendations, and index codes were added to attribute annotations to each sample. Clustering of the index-coded samples was performed on a cBot Cluster Generation System using the TruSeq PE Cluster Kit v4-cBot-HS (Illumina) according to the manufacturer’s instructions. After cluster generation, library preparations were sequenced on an Illumina platform. The expression levels of mRNAs identified by transcriptomic sequencing results were analyzed with reference to *Homo sapiens* GRCh38 genome. Genes with fold change ≥2 and false discovery rate < 0.01 were regarded as differentially expressed genes (DEGs). All the DEGs were used for annotation and enrichment analysis using the Kyoto Encyclopedia of Genes and Genomes (KEGG) and Gene Ontology (GO) databases, and significant enrichment was considered at a value of *p* < 0.05.

### 2.9. Statistical analysis

Data are expressed as the mean ± SD, and effects were considered significant if value of *p* < 0.05. SPSS 26.0 software was used for one-way ANOVA, following a *post hoc* analysis with the Duncan method. Histograms were constructed using Prism 9.1.1 (GraphPad Software Inc., San Diego, CA, United States).

## 3. Results

### 3.1. Plantaricin BM-1 inhibits the proliferation of CRC cells

**Figure 1:**
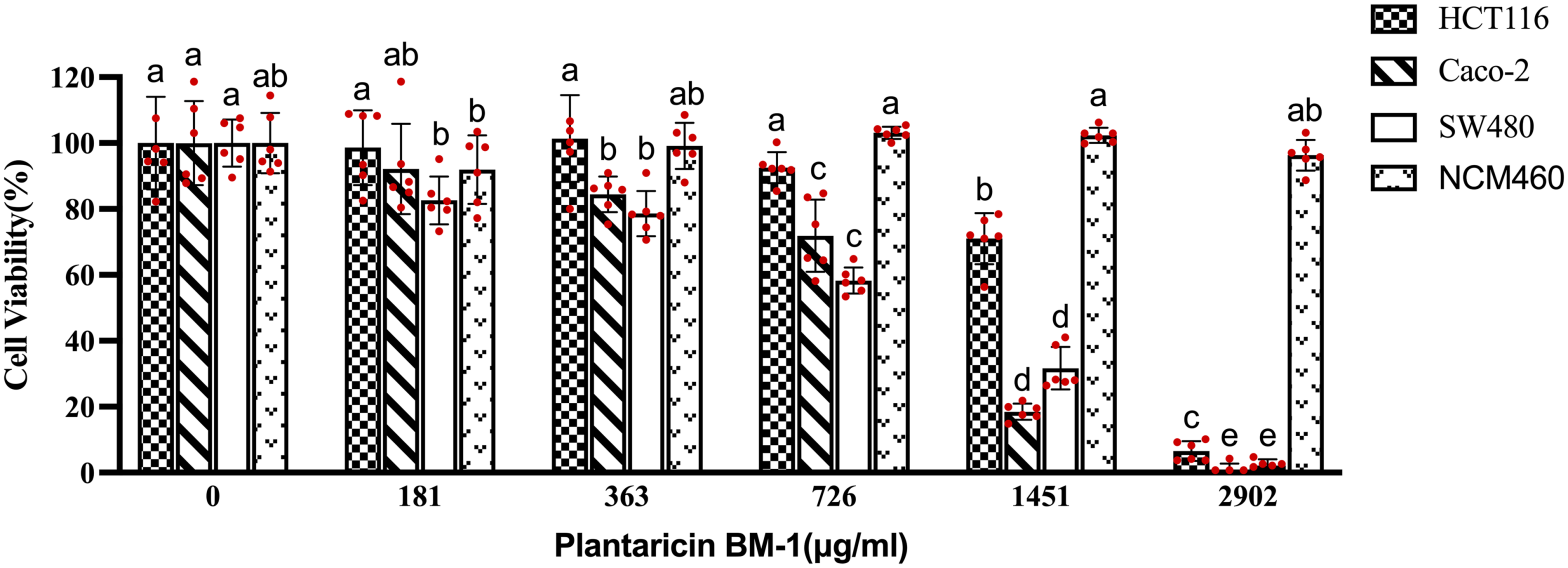
Effects of plantaricin BM-1 on cell viability in colorectal cancer (CRC) cells and normal intestinal epithelial NCM460 cells. The cells were treated with different concentrations of plantaricin BM-1 for 1 h and then, the cell survival rate (%) was determined by the CCK-8 kit. Each experiment was performed six times, and the results are presented in individual columns as the mean ± SD; (a–e) Different letters in bars indicate that means are significantly different at *p* < 0.05 according to one-way ANOVA in different plantaricin BM-1 concentration environments in the same cell line.

To investigate the anti-proliferative effect of plantaricin BM-1, a CCK-8 kit was used to quantify the viability of HCT-116, Caco-2, SW480, and NCM460 cells. As shown in Figure 1, treatment with plantaricin BM-1 significantly reduced CRC cell viability in a dose-dependent manner at 1 h compared to that in negative control. In contrast, plantaricin BM-1 showed no cytotoxicity against the normal colonic epithelial NCM460 cell line. The IC_50_ values of plantaricin BM-1 were 1,578, 819.9, and 757.9 μg/ml in HCT-116, Caco-2, and SW480 cells, respectively. Among these, SW480 cells appeared to be the most sensitive to the inhibitory effect of plantaricin BM-1. Incubation of SW480 cells with 181, 363, 726, 1,451, and 2,902 μg/ml plantaricin BM-1 led to significant cytotoxic effects and decrease of cell viability to 82.6 ± 7.24, 78.6 ± 6.84, 58.29 ± 3.99, and 31.69 ± 6.45, and 2.14 ± 1.89%, respectively, of the values in negative control cells. Compared to its cytotoxic action in SW480 cells, the inhibitory effect of plantaricin BM-1 on HCT-116 cells was weak. Even when the concentration of plantaricin BM-1 was 1,451 μg/ml, the survival rate of HCT-116 cells remained high, at 71.01% of the negative control value. Except for the normal colonic epithelial NCM460 cells, none of the three other cell lines survived after treatment with 2,902 μg/ml plantaricin BM-1. The cell viability assay results indicated that plantaricin BM-1 was cytotoxic to CRC cells, especially SW480 cells, and showed no toxicity toward the normal colonic epithelial NCM460 cells. Therefore, SW480 cells were selected for subsequent experiments.

### 3.2. Scanning electron microscopy of cell morphology changes after plantaricin BM-1 treatment

**Figure 2:**
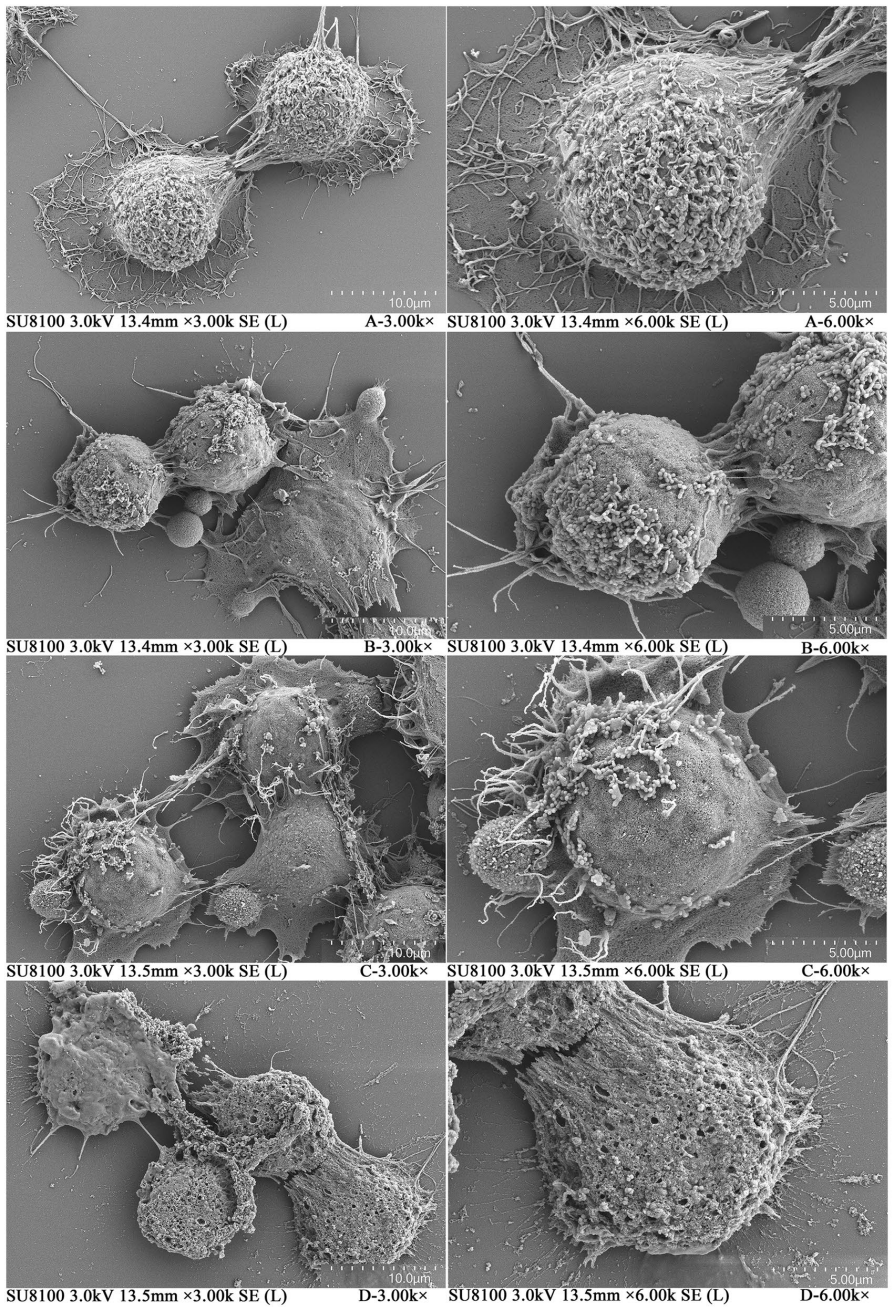
Scanning electron microscopy (SEM) of SW480 cells treated with plantaricin BM-1. Morphology of SW480 cells treated with different concentrations of plantaricin BM-1 for 1 h is illustrated as follows: **(A)** negative control, **(B)** 1/2 × IC_50_, **(C)** 1 × IC_50_, and **(D)** 2 × IC_50_.

Morphological changes in SW480 cells treated with different concentrations of plantaricin BM-1 for 1 h were examined by SEM. As shown in Figure 2A, in the negative control group without plantaricin BM-1 treatment, the cells were intact and surrounded by numerous microvilli. In contrast, the morphology of SW480 cells changed significantly after treatment with plantaricin BM-1 at 1/2 × IC_50_ and 1 × IC_50_: most of the microvilli vanished, and a few cells collapsed slightly (Figures 2B,C). In SW480 cells treated with 2 × IC_50_ plantaricin BM-1, microvilli disappeared completely, and vacuolization and membrane perforation were observed (Figure 2D). These changes indicated that plantaricin BM-1 had a concentration-dependent cytotoxic action on SW480 cells, which was in agreement with the CCK-8 results.

### 3.3. Plantaricin BM-1 induces apoptosis of SW480 cells

**Figure 3:**
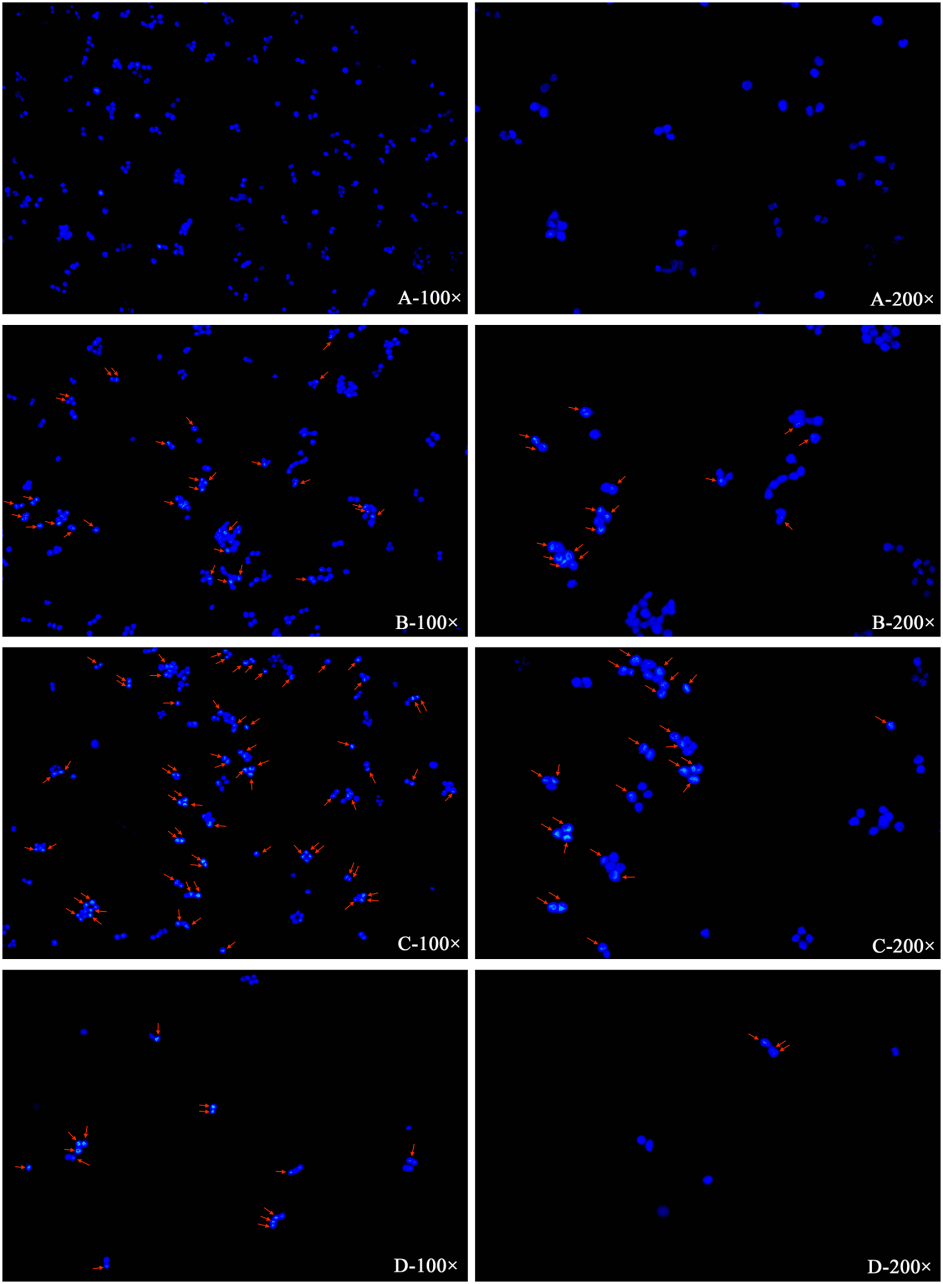
Induction of apoptosis in SW480 cells by plantaricin BM-1 revealed by fluorescence microscopy. SW480 cells were incubated for 1 h with various concentrations of plantaricin BM-1: **(A)** negative control, **(B)** 1/2 × IC_50_ plantaricin BM-1, **(C)** 1 × IC_50_ plantaricin BM-1, and **(D)** 2 × IC_50_ plantaricin BM-1. Then, the cells were stained with Hoechst33342 and photographed under a fluorescence microscope at magnifications of 100 and 200×. The arrow shows typical apoptotic cell features, such as chromatin condensation, cell contraction, and nuclear cracking.

After treatment with 1/2 × IC_50_, 1 × IC_50_, and 2 × IC_50_ of plantaricin BM-1 for 1 h, the morphology of SW480 cells was observed using Hoechst33342 staining under a fluorescence microscope. In the negative control group (Figure 3A), the cell bodies had a clear round nucleus and were intact, with weak fluorescence intensity. However, SW480 cells treated with plantaricin BM-1 (Figures 3B–D) were brighter and had condensed chromatin and nuclear fragmentation, which are characteristic features of apoptosis. The number of apoptotic cells increased in a dose-dependent manner, and in the 1 × IC_50_ plantaricin BM-1 treatment group, it was significantly higher than that in the 1/2 × IC_50_ plantaricin BM-1 treatment group. In addition, the number of viable cells in the 2 × IC_50_ plantaricin BM-1 treatment group decreased significantly. This indicated that plantaricin BM-1 induced a concentration-dependent pro-apoptotic effect.

**Figure 4:**
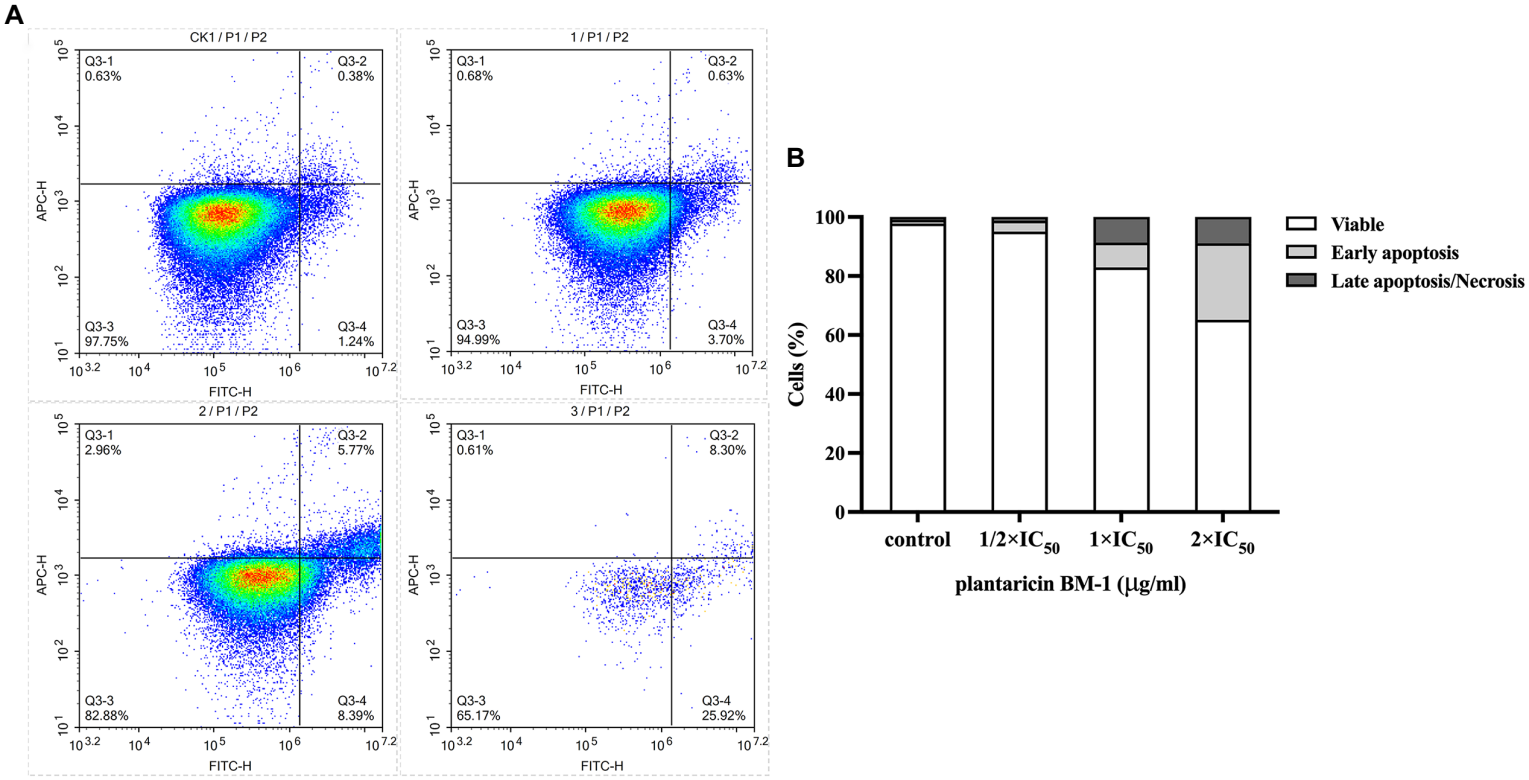
Quantitative analysis of cell apoptosis by flow cytometry with annexin V-FITC/PI double staining. **(A)** Flow cytometry was applied to analyze SW480 cell apoptosis after exposure to different concentrations of plantaricin BM-1 (CK1, control; 1, 1/2 × IC_50_ plantaricin BM-1; 2, 1 × IC_50_ plantaricin BM-1; and 3, 2 × IC_50_ plantaricin BM-1) for 1 h. **(B)** Histograms show relative cell number in four different states: live, early apoptosis, late apoptosis, and necrosis.

Apoptosis of SW480 cells treated with plantaricin BM-1 for 1 h was studied *via* flow cytometry with annexin V-FITC/PI double staining (Figure 4). After treatment with plantaricin BM-1 at 1/2 × IC_50_, 1 × IC_50_, and 2 × IC_50_, the apoptotic SW480 cells (including early and late apoptotic cells) comprised 4.33, 14.16, and 34.22%, respectively, of the total cells. This assay indicated that plantaricin BM-1 induced apoptosis of SW480 cells in a concentration-dependent manner, as determined by the increase in the signal detected in the first and fourth quadrants with an increase in the treatment dose (Figure 4). A total of 94.99% of the cells were still alive after treatment with 1/2 × IC_50_ of plantaricin BM-1. SW480 cells exposed to 1 × IC_50_ plantaricin BM-1 had a much larger cell population undergoing early apoptosis. A significant decline in cell viability and a drastic increase in apoptosis were observed in SW480 cells exposed to 2 × IC_50_ plantaricin BM-1.

Fluorescence microscopy and flow cytometry results indicated that the cytotoxicity of plantaricin BM-1 in SW480 cells was related to the promotion of apoptosis.

### 3.4. Plantaricin BM-1 activates caspase-3 and induces PARP-1 cleavage

**Figure 5:**
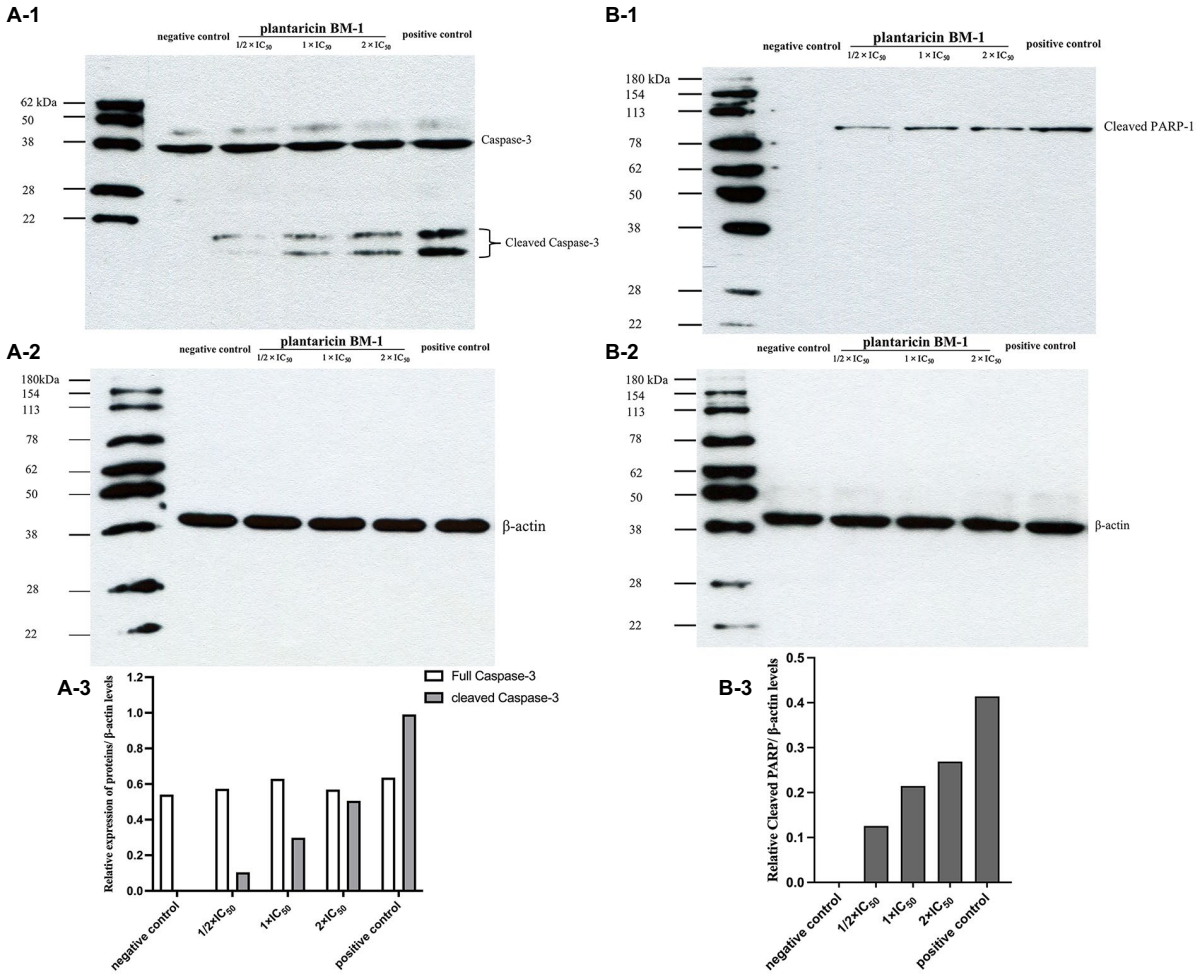
Western blot analysis of the levels of cleaved caspase-3 and cleaved PARP-1 after plantaricin BM-1 treatment. SW480 cells were treated with complete medium (negative control), plantaricin BM-1 (1/2 × IC_50_, 1 × IC_50_, or 2 × IC_50_), or 5-FU (positive control) for 1 h. **(A-1,A-3)** indicate the expression level of cleaved caspase-3. **(B-1,B-3)** indicate the expression level of cleaved PARP-1. Data were normalized to the level of β-actin protein expression, which was used as internal control (shown in **A-2** and **B-2**).

We used western blot analysis to determine the levels of cleaved caspase-3 and cleaved PARP-1 in SW480 cells cultured with or without plantaricin BM-1. Cells treated with 757.9 μg/ml 5-FU were used as the positive control. Data were normalized by the level of β-actin expression used as the internal control. Western blot results showed that expression levels of cleaved caspase-3 and cleaved PARP-1 were directly proportional to the concentration of plantaricin BM-1 used to treat SW480 cells (Figure 5). These results indicated that plantaricin BM-1 induced caspase-dependent apoptosis in SW480 cells.

### 3.5. Transcriptomic analysis of the effects of plantaricin BM-1 treatment on gene expression in SW480 cells

**Figure 6:**
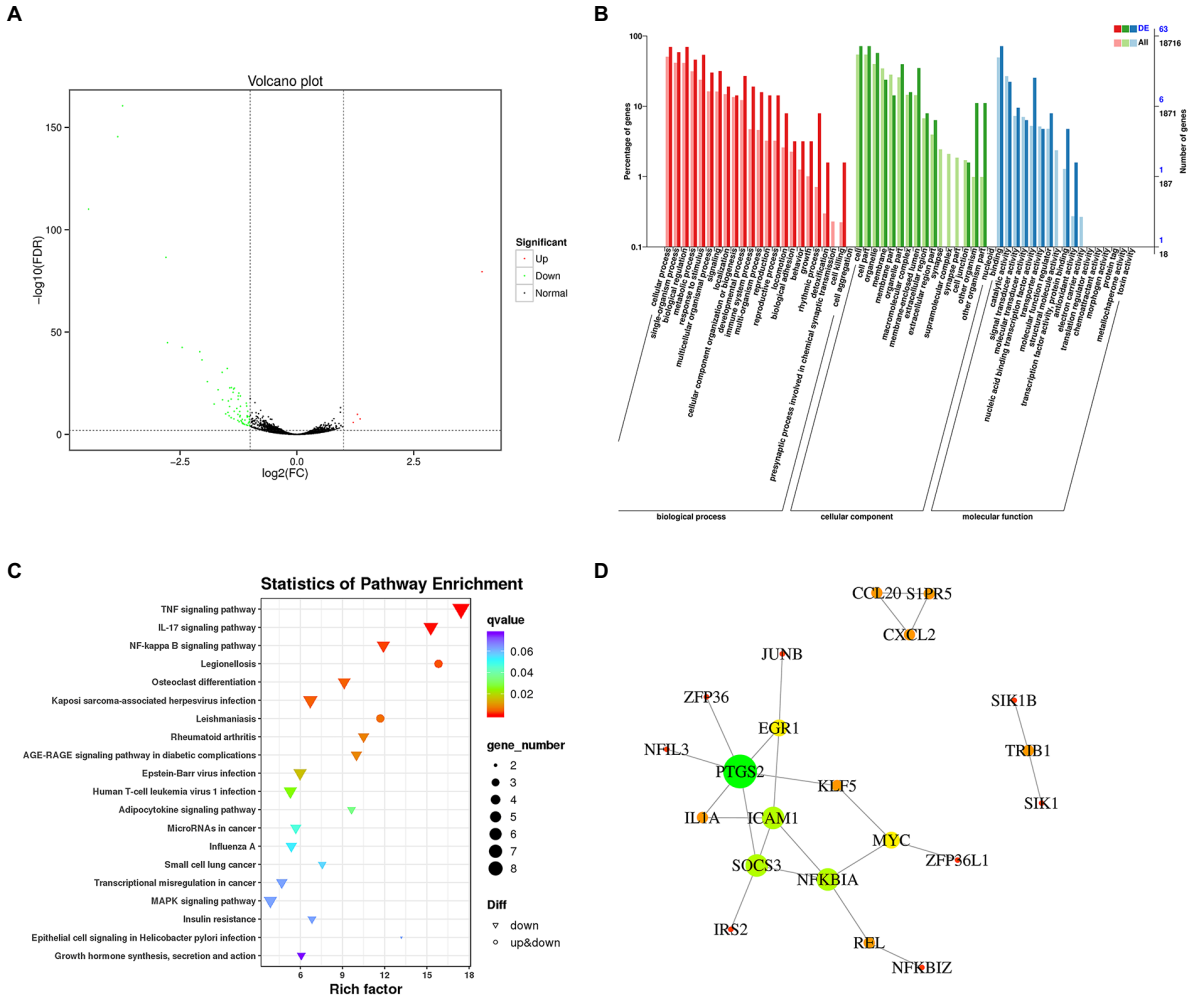
Transcriptome analysis of gene expression changes in SW480 cells after plantaricin BM-1 treatment. **(A)** Volcano plot. **(B)** GO enrichment analysis. **(C)** Kyoto Encyclopedia of Genes and Genomes (KEGG) pathway enrichment analysis. **(D)** Protein–protein interaction network. The nodes and lines represent proteins and interactions, respectively. The node size is proportional to the number of edges connected to the node. The node color represents the clustering coefficient (red = high clustering coefficient; green = low clustering coefficient). A total of 21 nodes and 23 edges were identified.

In this study, we showed that plantaricin BM-1 inhibited the proliferation of SW480 cells by promoting caspase-induced apoptosis. To systematically elucidate the mechanism underlying the suppressive action of plantaricin BM-1 on SW480 CRC cells, we used RNA sequencing transcriptome analysis (RNA-Seq) to analyze three biological replicates in each group of samples. As shown in the volcano plot (Figure 6A), 69 differentially expressed genes were screened from 23,152 transcriptome-annotated genes, of which 65 genes were downregulated, and four genes were upregulated (fold change ≥ 2; false discovery rate < 0.01).

Gene Ontology enrichment analysis was used to further analyze DEGs with a focus on biological processes (BP), cellular components (CC), and molecular functions (MF). As shown in Figure 6B, the classification based on BP demonstrated that DEGs were mainly related to cellular processes (63.77%), single-organism processes (52.17%), metabolic processes (53.62%), biological regulation (55.07%), and response to stimulus (46.38%). From the perspective of the CC category, DEGs were mainly associated with the cell (65.22%), cell part (65.22%), organelle (52.17%), membrane (21.74%), and membrane part (13.04%). With regard to MF, DEGs were mainly related to binding (65.22%) and catalytic activity (20.29%).

Based on the KEGG pathway database for enrichment analysis, DEGs in SW480 CRC cells were significantly enriched in 34 KEGG pathways (*p* < 0.05). The top 20 enrichment pathways with the lowest value of *p*s are shown in Figure 6C. In addition, we investigated the relevant pathways that have been documented to regulate the growth and apoptosis of colon cancer cells (Supplementary Figure 1), including the TNF signaling pathway (ko04668), NF-κB signaling pathway (ko04064), MAPK signaling pathway (ko04010), microRNAs in cancer (ko05206), and transcriptional misregulation in cancer (ko05202). These pathways and DEGs are listed in Table 1. Twenty-seven DEGs were significantly downregulated in these signaling pathways in response to plantaricin BM-1 treatment.

**Table 1:** Significantly enriched signaling pathways associated with CRC and the genes involved.

Pathway	Enrich_factor	Value of *p*	Down_genes	False discovery rate	Log2FC
TNF signaling pathway	17.43	1.26E−08	PTGS2	2.36E−08	−1.345696228
			CXCL2	2.82E−05	−1.013569651
			ICAM1	2.46E−05	−1.080316693
			NFKBIA	6.73E−05	−1.006230115
			CCL20	3.68E−05	−1.052786073
			TNFAIP3	9.22E−11	−1.522580166
			JUNB	1.82E−23	−1.435749373
			SOCS3	1.37E−09	−1.052087258
NF-kappa B signaling pathway	11.91	5.60E−05	PTGS2	2.36E−08	−1.345696228
			CXCL2	2.82E−05	−1.013569651
			ICAM1	2.46E−05	−1.080316693
			NFKBIA	6.73E−05	−1.006230115
			TNFAIP3	9.22E−11	−1.522580166
MAPK signaling pathway	3.87	8.74E−03	IL1A	5.97E−06	−1.154147302
			DUSP1	6.87E−33	−1.489214608
			NR4A1	2.55E−161	−3.729128244
			MYC	2.54E−06	−1.158861570
			DUSP5	1.15E−17	−1.589627729
MicroRNAs in cancer	5.68	5.12E−03	PTGS2	2.36E−08	−1.345696228
			MYC	2.54E−06	−1.15886157
			PIM-1	3.12E−21	−1.364514968
			IRS2	1.18E−05	−1.117769098
Transcriptional misregulation in cancer	4.68	1.01E−02	NR4A3	2.97E−43	−2.454790071
			MYC	2.54E−06	−1.158861570
			NFKBIZ	1.32E−45	−2.769516860
			REL	2.18E−09	−1.219135810

To gain a systematic insight into the signaling networks involved in the induction of apoptosis and resulting growth inhibition in SW480 cells following exposure to plantaricin BM-1, we combined the results of differential expression analysis and the interaction pairs collected in the STRING database to construct a protein interaction network of SW480 cell DEGs by using Cytoscape software. The protein–protein interaction network of DEGs is shown in Figure 6D. A total of 21 nodes and 23 edges were identified. Three highly connected clusters were obtained from the cluster analysis (Supplementary Table 1). These findings revealed complicated mechanisms underlying the effects of plantaricin BM-1 in SW480 cells.

## 4. Discussion

Plantaricin BM-1 is a class IIa bacteriocin that, similarly to other known class IIa bacteriocins, has antimicrobial activity against various Gram-positive and Gram-negative bacteria (Zhang et al., 2013). In this study, we found for the first time that plantaricin BM-1 inhibited SW480 CRC cell growth by reducing proliferation and inducing apoptosis.

Our results showed that plantaricin BM-1 specifically acted on cancer cells and was not toxic to normal cells. Moreover, the cytotoxicity of this bacteriocin in SW480 cells was stronger than that in the other two colon cancer cell lines, indicating that SW480 cells were particularly susceptible. In agreement with our present data, the antimicrobial peptides M2163 and M2386 were also significantly cytotoxic to SW480 and Caco-2 cells but not to normal cells. The MTT assay gives an IC_50_ of SW480 cells treated with either peptide m2163 or m2386 for 24 h was 40 μg/ml (Tsai et al., 2015). Compared with our results (Figure 1), plantaricin BM-1 could kill SW480 cells after 1 h treatment, although the IC_50_ was higher at 757.9 μg/ml. In addition, according to a previous report, nisin decreased the viability of SW480 cells in a dose-dependent manner. Survival rates of 42.94, 41.77, 79.22, 130.6, 129, and 132.1% were detected after treatment with nisin at different concentrations (3,000, 2,500, 1,000, 750, 325, and 250 μg/ml, respectively) for 24 h (Jaye et al., 2022). Our results (Figure 1) showed that the IC_50_ value of plantaricin BM-1 in relation to its effect on SW480 cells was 757.9 μg/ml, indicating that the inhibitory effect of plantaricin BM-1 was better than that of nisin. Thus, plantaricin BM-1 specifically inhibited SW480 CRC cell proliferation at lower concentrations and over a shorter period of time.

To further investigate the mechanism underlying the cytotoxic action of plantaricin BM-1 on SW480 cells, SEM was used to observe changes in cell morphology and cell membrane surface after treatment. As was demonstrated by Mejía-Caballero et al. (2021), SW480 cells treated with the bacteriocin KL15 had pores in the membrane after treatment, and their microvilli disappeared, which is similar to the SEM images of plantaricin BM-1 after 2 × IC_50_ treatment (Figure 2D). These results indicated that similar to KL15, 2 × IC_50_ plantaricin BM-1 may also killed SW480 cells through necrosis. Similarly, the microvilli of cancer cells disappeared, and the membrane surface became smooth after treatment with the anticancer peptide LS10, as observed by SEM (Baindara et al., 2017), which was consistent with that after treatment with 1/2 × IC_50_ and 1 × IC_50_ plantaricin BM-1 (Figures 2B,C). This indicated that plantaricin BM-1 does not induce cell death through necrosis at lower concentrations, but only affected SW480 cell morphology, while the cell membrane remained intact.

Apoptosis is considered to be one of the most promising ways to inhibit cancers (Carneiro and El-Deiry, 2020). In this study, we focused on the suppression of SW480 cell treated with plantaricin BM-1 *via* induction of apoptosis. In previous studies, Hoechst33342 staining was used to observe the increase in fluorescence intensity of apoptotic cells, as well as aggregation and fragmentation of the nuclei (Goud et al., 2020; Wu et al., 2020; Huang et al., 2021; Alafnan et al., 2022). This was similar to our results (Figure 3) using Hoechst33342 staining, which demonstrated that after receiving plantaricin BM-1 for 1 h, the initiation of the apoptosis process *via* enhanced chromatin condensation and the number of cells with condensed nuclei of high fluorescence intensity was increased dose-dependently. And the number of apoptotic cells increased in a dose-dependent manner. Compared with fluorescence microscopy, the flow cytometry analysis provides better qualitative and quantitative analyses of apoptotic cells (Pereira et al., 2021). The bacteriocin m2163 has been demonstrated to induce SW480 cell apoptosis by annexin V/PI staining and flow cytometry analysis after 24 h treatment (Tsai et al., 2015). Compared with m2163, plantaricin BM-1 could induce apoptosis in SW480 cells in a shorter time. Using this method, we showed that plantaricin BM-1 treatment of SW480 cells for 1 h also induced apoptosis. And the corresponding apoptosis rate at 1 × IC_50_ concentration was 14.16% (Figure 4). This was mirrored by fluorescence microscopy results (Figure 3).

Caspase-3 protein is key regulator of the execution of apoptosis, which is responsible for the actual cleavages of intracellular proteins, such as PARP-1, resulting in apoptotic cell death (Yang et al., 2019; Zhou et al., 2021; Asadi et al., 2022). The observed cleavage of caspase-3 and cleavage of PARP-1 following plantaricin BM-1 treatment indicated that plantaricin BM-1 can induce caspase-dependent apoptosis in SW480 cells (Figure 5). This is similar to the mechanism of apoptosis induced by deoxypodophyllotoxin in SW480. Moreover, deoxypodophyllotoxin has also been confirmed to mediate the intrinsic mitochondrial pathway to induce CRC cell apoptosis by increasing the level of pro-apoptotic protein Bax and decreasing the level of anti-apoptotic protein Bcl-xL (Gamage et al., 2019), which was not involved in our study and can be carried out in subsequent studies. In addition, our findings suggested that the degree of caspase-3 cleavage after treatment with 1 × IC_50_ plantaricin BM-1 was significantly lower than that after treatment with 5-FU at the same concentration. However, CCK-8 results showed that cell viability after plantaricin BM-1 treatment was significantly lower than that after 5-FU treatment (data not shown). This indicates that bacteriocin plantaricin BM-1 may induce SW480 cell death through pathways other than the caspase-dependent apoptosis pathway, which needs to be further studied.

We then applied transcriptomic technology and bioinformatics analysis to comprehensively reveal the molecular mechanism of plantaricin BM-1 effects on SW480 cells. Notably, our data suggested that plantaricin BM-1 triggered apoptosis and suppressed the viability of SW480 cells by regulating multiple signaling pathways. Changes in genes encoding the TNF signaling pathway components were the most significant. The TNF signaling pathway mediates apoptosis in SW480 cells. It has been reported that boric acid treatment activates the TNF signaling pathway, which, in turn, induces apoptosis in SW480 cells, and the qRT-PCR results showed that the expression levels of *TNF* and *TNFSF8* genes in the treatment group were significantly upregulated (Sevimli et al., 2022). However, the expression levels of these two genes were not significantly altered in our transcriptomic analysis. Our results showed that among the DEGs related to the TNF signaling pathway, *TNFAIP3* expression was most significantly downregulated in response to plantaricin BM-1 treatment. The TNFAIP3 protein disrupts the recruitment of death domain signaling molecules TRADD and RIP to receptor signaling complexes and protects cells from TNF-induced apoptosis (Martens and van Loo, 2020). In addition, the knockdown of *TNFAIP3* promoted TNF-induced apoptosis (Priem et al., 2019; Mulla and Venkatraman, 2022). As shown in previous studies, aspirin can induce apoptosis of CRC cells by degrading NFKBIA, leading to nuclear translocation of the NF-κB complex and activation of the NF-κB pathway (Prescott and Cook, 2018). Similarly, the expression of NFKBIA was significantly reduced in our study, and KEGG enrichment analysis revealed changes in the NF-κB signaling pathway. This indicates that plantaricin BM-1 may also induce apoptosis in SW480 cells by activating the NF-κB signaling pathway. The MAPK signaling pathway plays a regulatory role in cell survival, growth, and apoptosis (Yue and López, 2020). Asterosaponins induced apoptosis by inactivating the ERK1/2 MAPK pathway and downregulating *MYC* (Thao et al., 2014). Indeed, significant downregulation of *MYC* and alterations in MAPK signaling pathways were observed in our results. miRNAs play an important role in promoting or inhibiting cancer cell proliferation and apoptosis by regulating oncogenes or tumor suppressor genes (Zhi et al., 2018). Oncogene PIM-1 is usually overexpressed in colon cancer (Zhang et al., 2010). Knockdown or downregulation of PIM-1 was shown to inhibit tumor proliferation and induce mitochondria-mediated apoptosis by activating caspase-9 (Weirauch et al., 2013; Rathi et al., 2021). Our transcriptomic results also revealed a decrease in the *PIM1* gene expression. These results provide evidence that plantaricin BM-1 activates multiple pathways to induce apoptosis.

## 5. Conclusion

In this study, we demonstrated for the first time that plantaricin BM-1 shows no cytotoxicity against the normal colonic epithelial NCM460 cell line, but inhibits SW480 CRC cell proliferation. The IC_50_ was 757.9 μg/ml. Besides, plantaricin BM-1 induces apoptosis through the caspase-3 pathway *in vitro*. Furthermore, using transcriptomic and bioinformatic analyses, we explored possible mechanisms underlying the cytotoxic effects of this bacteriocin. We found that 65 genes were downregulated and four were upregulated. KEGG analysis showed that the dysregulation of the TNF, NF-kB, and MAPK signaling pathways, altered levels of cancer-related miRNAs, and changes in the expression of genes mediating transcriptional misregulation in cancer were likely involved in the biological mechanism by which plantaricin BM-1 induced apoptosis in SW480 cells. This spectrum of biological activity shows that plantaricin BM-1 should be studied further as a promising anti-CRC compound.

## Data availability statement

The datasets presented in this study can be found in online repositories. The names of the repository/repositories and accession number(s) can be found at: NCBI, https://www. ncbi.nlm.nih.gov/sra/PRJNA910522.

## Author contributions

YX and HW: conceptualization and methodology. HW and ZB: software. HW and JZ: validation. JJ, XP, and YH: resources. YX: data curation and writing—review and editing. HW: writing—original draft preparation. HZ and YX: supervision. All authors contributed to the article and approved the submitted version.

## Funding

This work was supported by the Research project of Beijing Municipal Commission of Education (KM201810020016).

## Conflict of interest

The authors declare that the research was conducted in the absence of any commercial or financial relationships that could be construed as a potential conflict of interest.

## Publisher’s note

All claims expressed in this article are solely those of the authors and do not necessarily represent those of their affiliated organizations, or those of the publisher, the editors and the reviewers. Any product that may be evaluated in this article, or claim that may be made by its manufacturer, is not guaranteed or endorsed by the publisher.

## Footnotes

^1^ www.biocloud.net
