# Supplementary material for: Plantaricin BM-1 decreases viability of SW480 human colorectal cancer cells by inducing caspase-dependent apoptosis
Source: Front Microbiol. 2023 Jan 4;13:1103600. doi: 10.3389/fmicb.2022.1103600 (PMC9845772; doi:10.3389/fmicb.2022.1103600)
Supplement: Supplementary file 1 [file Data_Sheet_1.docx]

Supplementary Material


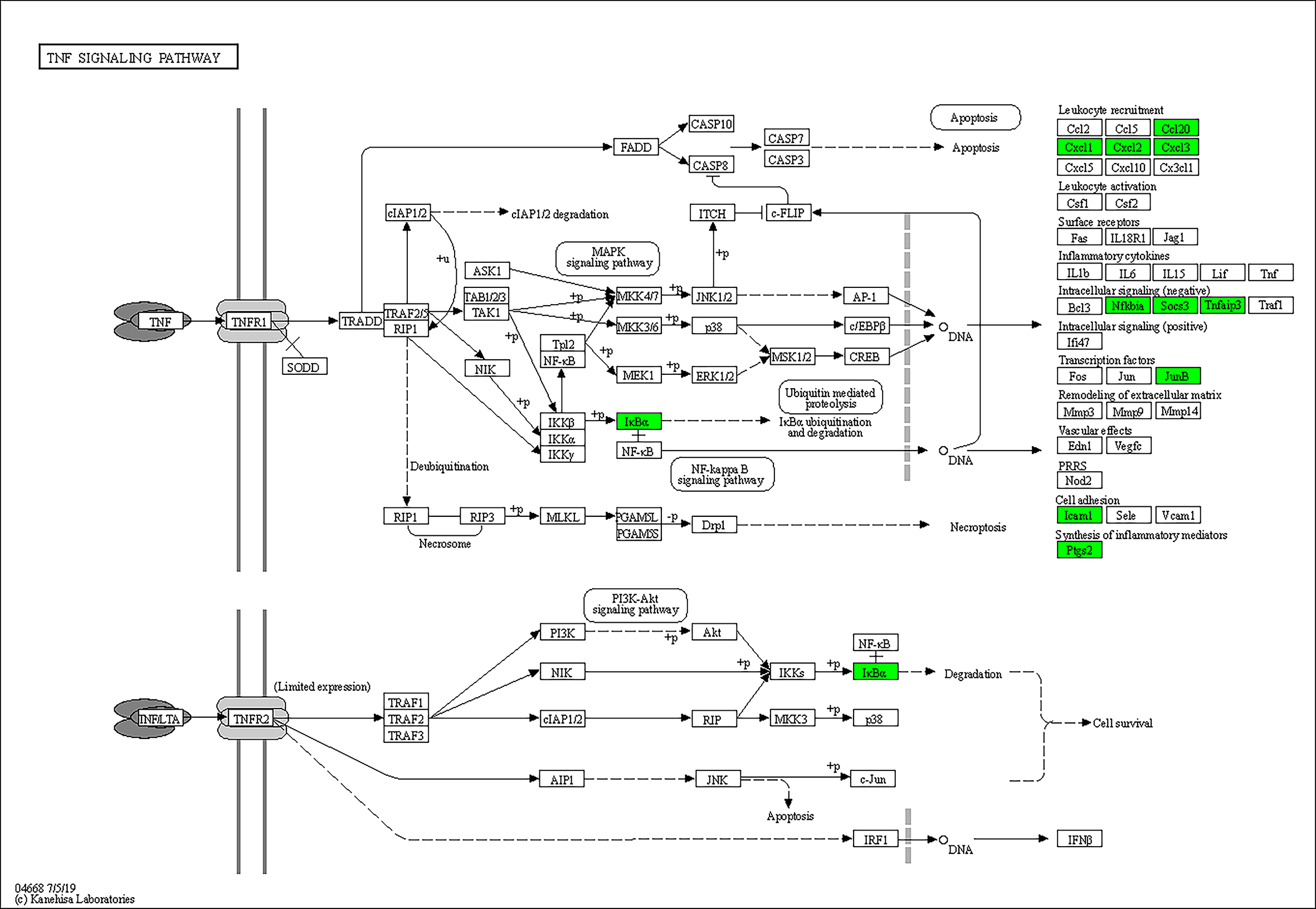

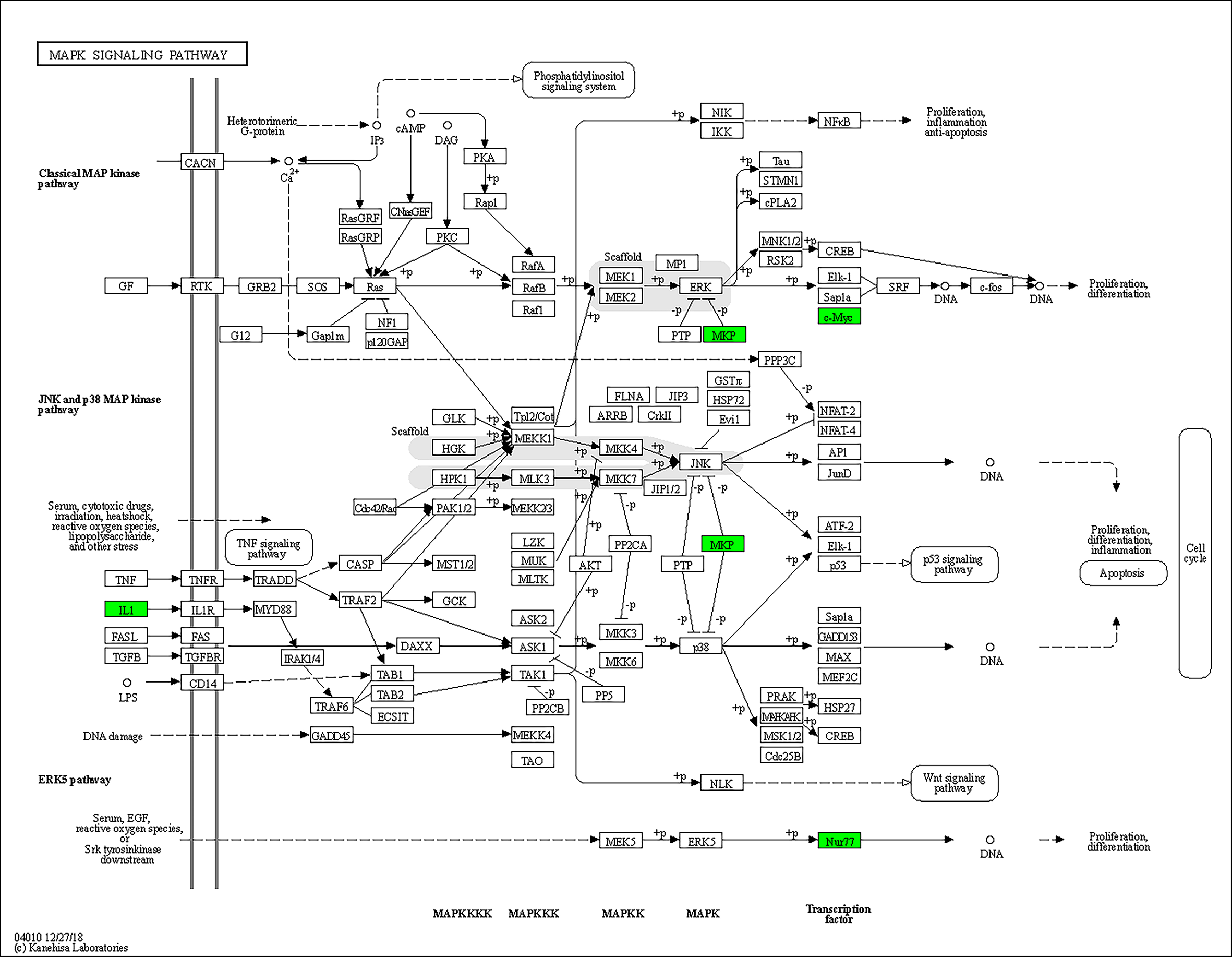


**
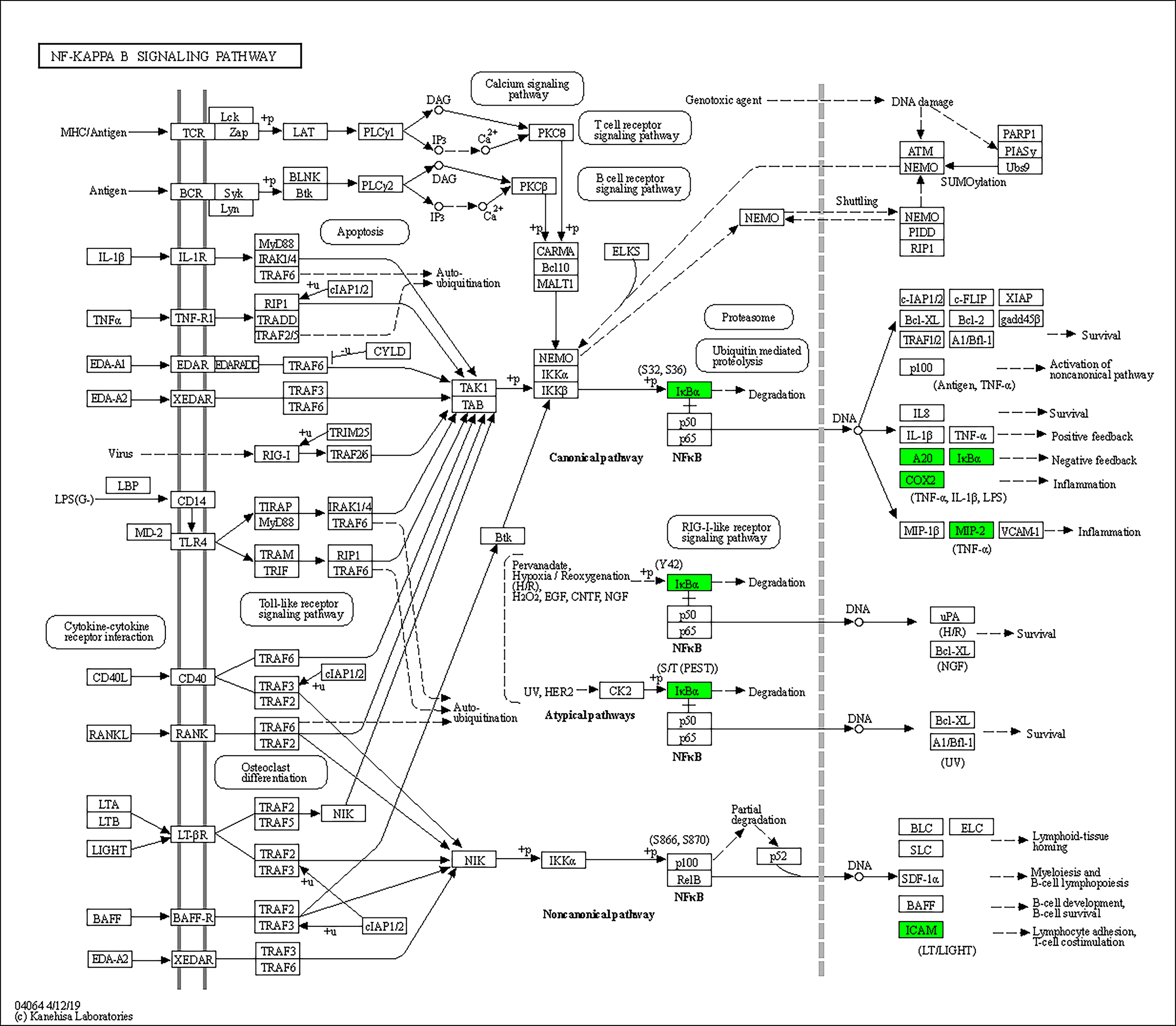
**

**
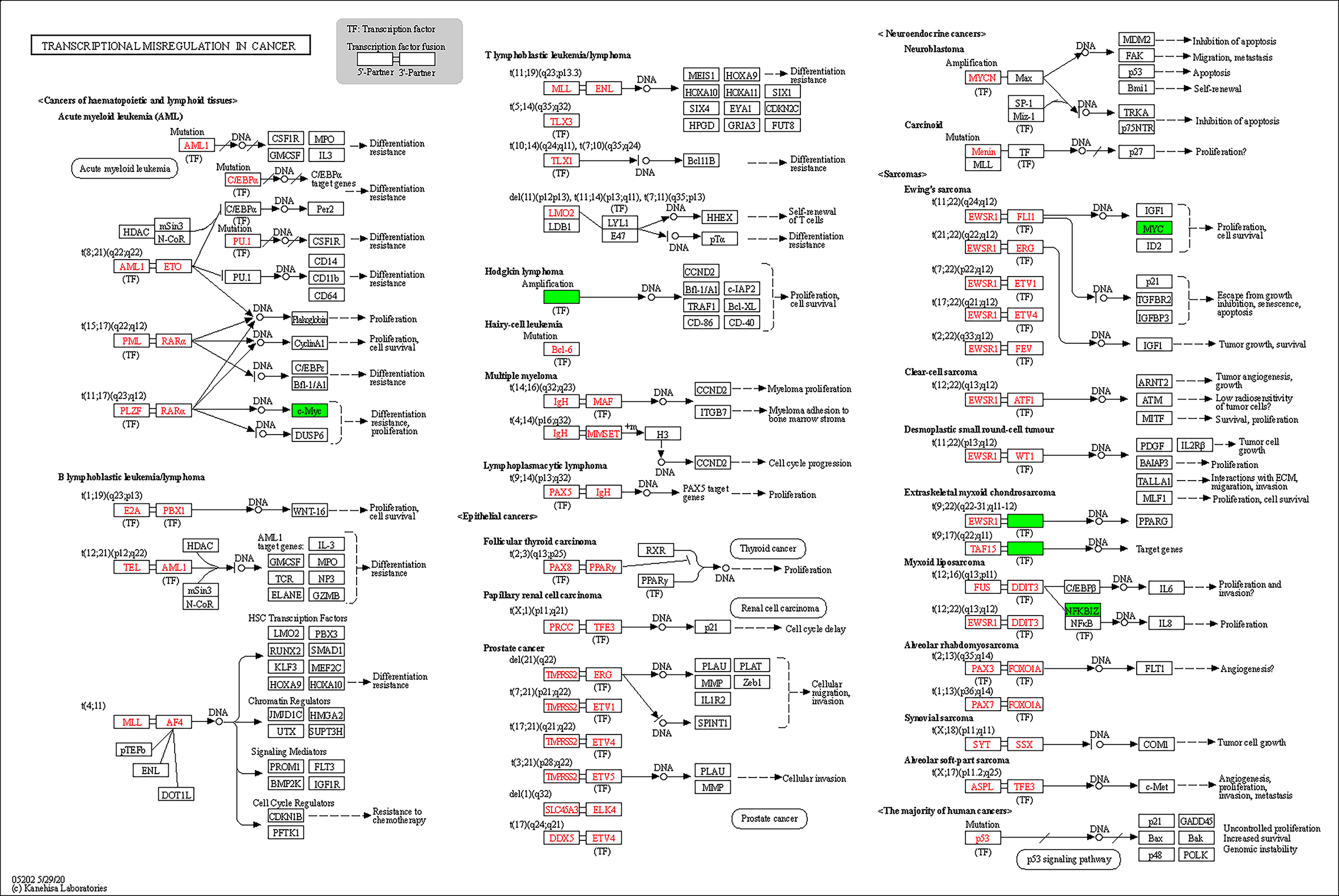
**

**
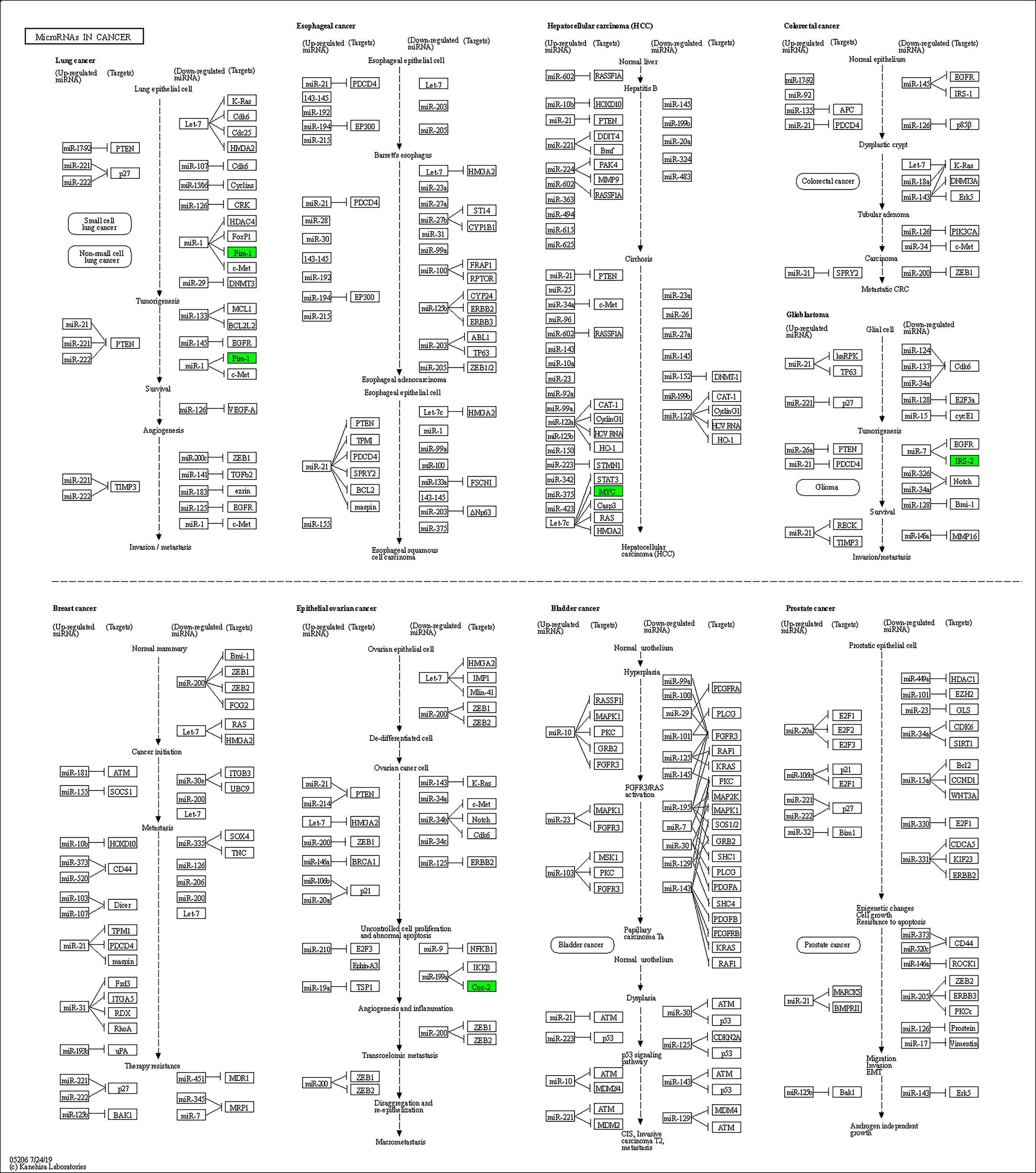
**

**Supplementary Figure 1.** KEGG pathway annotation diagram. KEGG enrichment analysis was used to screen the annotation map of the signaling pathways involved in the growth and apoptosis of colon cancer cells.

**Supplementary Table 1.** Degree of connection between proteins in the protein-protein interaction network analysis.

|  | **Edges** | **Gene ID** | **Protein symbol** | **Gene ID** | **Protein symbol** | **Mode(Degree)** |
| --- | --- | --- | --- | --- | --- | --- |
| cluster 1 | 1 | ENSG00000100906 | NFKBIA | ENSG00000162924 | REL | binding(951); reaction(951); inhibition(531); catalysis(167) |
|  | 2 | ENSG00000100906 | NFKBIA | ENSG00000136997 | MYC | reaction(900) |
|  | 3 | ENSG00000136997 | MYC | ENSG00000185650 | ZFP36L1 | inhibition(900) |
|  | 4 | ENSG00000184557 | SOCS3 | ENSG00000185950 | IRS2 | inhibition(805) |
|  | 5 | ENSG00000073756 | PTGS2 | ENSG00000115008 | IL1A | activation(579); expression(549); inhibition(549) |
|  | 6 | ENSG00000128016 | ZFP36 | ENSG00000073756 | PTGS2 | expression(469) |
|  | 7 | ENSG00000184557 | SOCS3 | ENSG00000100906 | NFKBIA | binding(452) |
|  | 8 | ENSG00000184557 | SOCS3 | ENSG00000090339 | ICAM1 | expression(446); inhibition(446) |
|  | 9 | ENSG00000073756 | PTGS2 | ENSG00000165030 | NFIL3 | expression(433); inhibition(433) |
|  | 10 | ENSG00000073756 | PTGS2 | ENSG00000120738 | EGR1 | expression(433); activation(433) |
|  | 11 | ENSG00000115008 | IL1A | ENSG00000090339 | ICAM1 | activation(433) |
|  | 12 | ENSG00000100906 | NFKBIA | ENSG00000090339 | ICAM1 | activation(429) |
|  | 13 | ENSG00000136997 | MYC | ENSG00000102554 | KLF5 | activation(426) |
|  | 14 | ENSG00000184557 | SOCS3 | ENSG00000073756 | PTGS2 | activation(416) |
|  | 15 | ENSG00000073756 | PTGS2 | ENSG00000102554 | KLF5 | expression(416) |
|  | 16 | ENSG00000090339 | ICAM1 | ENSG00000120738 | EGR1 | expression(416) |
|  | 17 | ENSG00000162924 | REL | ENSG00000144802 | NFKBIZ | binding(175) |
|  | 18 | ENSG00000171223 | JUNB | ENSG00000120738 | EGR1 | Inhibition(156) |
| cluster 2 | 19 | ENSG00000173334 | TRIB1 | ENSG00000142178 | SIK1 | binding(177); catalysis(177); ptmod(177); reaction(177) |
|  | 20 | ENSG00000173334 | TRIB1 | ENSG00000275993 | SIK1B | activation(177) |
| cluster 3 | 21 | ENSG00000081041 | CXCL2 | ENSG00000115009 | CCL20 | catalysis(903); reaction(903) |
|  | 22 | ENSG00000180739 | S1PR5 | ENSG00000081041 | CXCL2 | catalysis(900); reaction(900) |
|  | 23 | ENSG00000180739 | S1PR5 | ENSG00000115009 | CCL20 | catalysis(900); reaction(900) |
